# Ionization of 2‐ and 4(5)‐Nitroimidazoles Radiosensitizers: A *“Kinetic Competition”* Between NO_2_ and NO Losses

**DOI:** 10.1002/cphc.202100629

**Published:** 2021-10-12

**Authors:** Mauro Satta, Anna Rita Casavola, Antonella Cartoni, Mattea Carmen Castrovilli, Daniele Catone, Jacopo Chiarinelli, Stefano Borocci, Lorenzo Avaldi, Paola Bolognesi

**Affiliations:** ^1^ Istituto per lo Studio dei Materiali Nanostrutturati (ISMN-CNR) Dipartimento di Chimica Università degli studi di Roma La Sapienza P.le Aldo Moro 5 00185 Roma Italy; ^2^ Istituto di Struttura della Materia (ISM-CNR), Area della Ricerca di Roma 1 Via Salaria Km 29,300 00016 Monterotondo Scalo (RM) Italy; ^3^ Dipartimento di Chimica Università degli studi di Roma La Sapienza Pl.e Aldo Moro 5 00185 Roma Italy; ^4^ Istituto di Struttura della Materia (ISM-CNR), Area della Ricerca di Roma 2 Via del Fosso del Cavaliere 10 00133 Roma Italy; ^5^ Dipartimento per l'Innovazione nei Sistemi Biologici, Agroalimentari e Forestali (DIBAF) Università della Tuscia Viterbo Italy

**Keywords:** rate coefficient, radiotherapy, high-energy density materials, variational transition state theory, density functional calculations

## Abstract

Nitroimidazoles are a class of chemicals with a remarkable broad spectrum of applications from the production of explosives to the use as radiosensitizers in radiotherapy. The understanding of thedynamics of their fragmentation induced by ionizing sources is of fundamental interest. The goal of this work is to theoretically investigate the *kinetic competition* between the two most important decomposition channels of 2, 4 and 5‐Nitroimidazole cations: the NO and NO_2_ losses. The calculated rate constants of the two processes are in very good agreement with the experimental Photoelectron‐Photoion Coincidence (PEPICO) branching ratio. This study solves the intriguing and theoretically unexplained experimental observation that 2‐Nitroimidazole, at variance with the other two regio‐isomers is a source for only NO at low energies (<12.76 eV). This is a key point for biomedical application of the nitroimidazoles, because NO is the vasodilator that favors the reoxigenation of hypoxic tumor tissues.

## Graphical abstract

**The kinetic competition between the NO and NO_2_** loss channels in 2‐Nitroimidazole ions favours the former channel at low energies (<12.76 eV). Thus, at these energies, 2‐Nitroimidazole is an exclusive NO source, unlike 4 and 5‐Nitroimidazole isomers. This is important for biomedical applications since NO is a vasodilator that favors oxygenation of tissues.

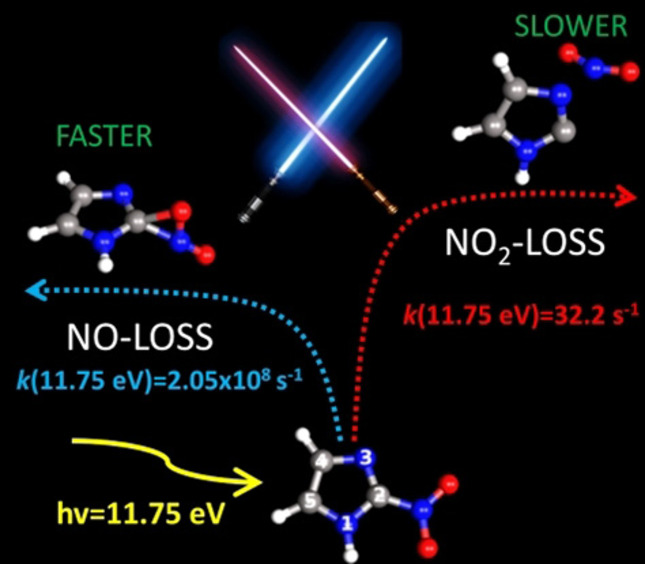

Nitroimidazoles (NIM) and their derivatives have numerous applications. They are attractive high‐energy density materials (HEDM) because during their decomposition the large amount of energy stored in their chemical bonds is released.^[1]^ In medicine,^[2]^ among others, they are used as radiosensitizing agents in radiotherapy enhancing the effect of radiation damage, though suffering from dose‐limiting neurotoxicities.^[3]^ NIM are classified as oxygen‐mimetic compounds since they increase the sensitivity of cells of solid hypoxic tumors to radiotherapy treatment. It has been observed that radiation induced decomposition of NIM releases NO and/or NO_2_. These molecules, which share with oxygen the radical nature, may act via a twofold mechanism.^[4]^ On the one hand, the radicals fix dangling bonds in radiation damaged DNA, reducing its natural repairing capability. On the other hand, NO, being a vasodilator, can favor the re‐oxygenation of the tumor tissues.^[5]^ Moreover, also drugs based on NIM isomers, like metronidazole and misonidazole, which release HNO_2_ in their fragmentation, indirectly can produce NO through the reduction of nitrite NO_2_^−^ into nitric oxide via reactions with several heme‐containing proteins, in particular in hypoxic states.^[6]^ In this framework, the physical chemistry studies of the NIM compounds, their fragmentation by different ionization sources and reactivity is of paramount importance.^[7]^ For this reason, the pathways leading to NO and NO_2_ losses after ionization of nitroimidazoles have been extensively investigated.[^4^, ^8^] However, the energetic of the NO_2_ loss channel (Figure 1) from the 2NIM ionized molecule, leading to the ion at m/z=67 (67^+^) with ring structure, remains under debate.

**Figure 1:**
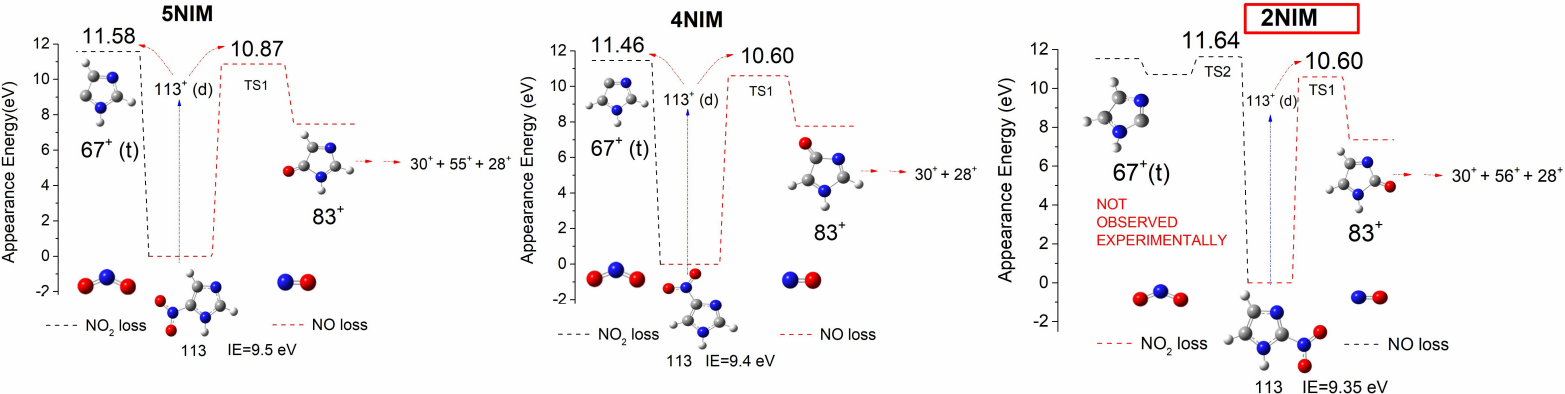
Scheme of the potential energy levels for the NO and NO_2_ loss channels of the three nitroimidazole cations (C_3_H_3_N_3_O_2_^+^).^[8b]^ The theoretical appearance energies (AE_th_) in eV at the CCSD/6‐311++G** level of theory of ions 83^+^ (NO loss) and 67^+^ (NO_2_ loss) are shown. All fragment ions are observed experimentally and the AEs_exp_ agree with the theoretical values, apart for the ion 67^+^ coming from 2NIM detected experimentally only above 12.76 eV. Ions 30^+^, 55^+^, 28^+^ and 56^+^ are produced in the subsequent fragmentation of ion 83^+^.^[8b]^ IE is the ionization energy.

Indeed, the experimental appearance energy of this ion, AE_exp_(67^+^)=12.76±0.06 eV^[8b]^ does not match with the calculated one, AE_th_(67^+^)=11.64 eV, obtained at the CCSD/6‐311++G** level of theory on geometries optimized at the B3LYP/6‐311++G** level of theory (Figure 1). Consequently, 2NIM appears to be only a source of NO (AE_exp_(83^+^)=10.86±0.02 eV and AE_th_(83^+^)=10.60 eV) at energies lower than 12.76 eV, through the nitro‐nitrite isomerization.^[9]^

On the other hand, in the 4 and 5NIM molecules, that coexist in a tautomeric equilibrium in the gas phase,^[10]^ the experimental and theoretical AE values of the NO_2_ loss channel show a good agreement, being AE_exp_(67^+^)=11.70±0.14 eV for the 4 and 5NIM mixture and AE_th_(67^+^)=11.46 eV and 11.58 eV for 4 and 5NIM, respectively (Figure 1).^[8b]^

Different arguments have been proposed to explain this discrepancy in the 2NIM. Recently, Meißner et al.^[11]^ reported that the NO_2_ loss channel occurs after the opening of the imidazole ring and obtained a theoretical value of ΔG_298_=12.77 eV for this process, at the M06‐2x/aug‐cc‐PVTZ level of theory. This value satisfactorily compares with their AE_exp_(67^+^)=13.00±0.05 eV obtained in an electron ionization experiment.

However, the most important and challenging question, concerning why the 2NIM parent ion does not follow the minimum energy path, i. e., the break of C2−NO_2_ bond, remains open. Indeed, we verified, at the M06‐2x/aug‐cc‐PVTZ level of calculations, that the channel with the closed ring (right part of Figure 2) – i. e., the channel studied through all the present work at the B3LYP/6‐311++G** level – represents a lower energy path with respect to the one where the ring opens (left part of Figure 2). Since the energy range considered in this work is up to 13.5 eV, we did not consider the kinetic aspects of the channel leading to an open ring, which is populated at energies larger than 13.49 eV (Figure 2).

**Figure 2:**
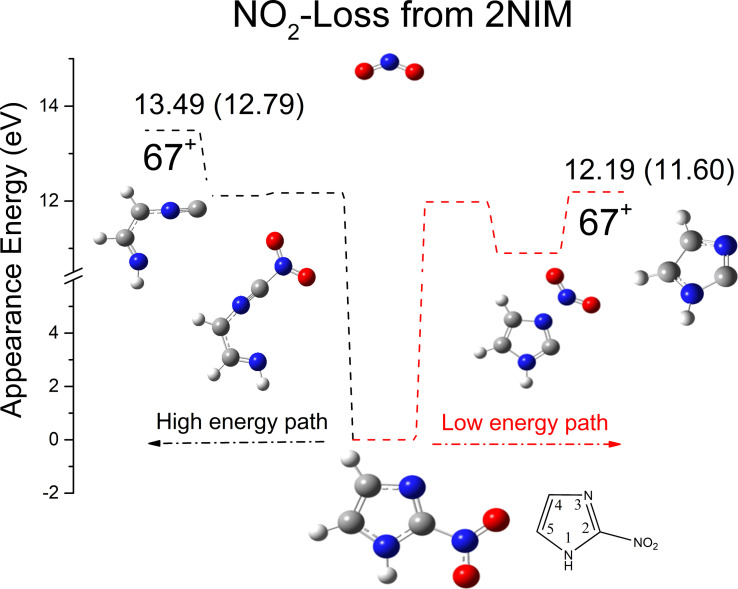
AE_th_ in eV, calculated at the M06‐2x/aug‐cc‐PVTZ level of theory, for the production of ions 67^+^ from 2NIM via the opening of the ring preceding the NO_2_ loss (left side in black) and from the breaking of the C2−NO_2_ bond (right side in red). The ΔG° values are reported in brackets.

The analysis of the energetic and structural data as well as the Mulliken charge analysis of the NO_2_ group^[12]^ and the dipole moment at the B3LYP/6‐311++G** level of theory in the three neutral and charged isomers (see Table S1 in Supporting Information and Figure S1) do not help in explaining the higher AE_exp_ of the 67^+^ ion in 2NIM with respect to 4(5)NIM. On the basis of these analysis and previous considerations^[8b]^ this work focuses on the fundamental kinetic aspects of the photo fragmentation,^[13]^ calculating the rate coefficient, *k*(E), of these different processes to establish the relative abundance of the corresponding fragments. In particular, the *kinetic competition* of the two pathways under investigation, NO versus NO_2_ loss has been studied as function of the energy. The microcanonical *k*(E) for the two channels (Figure 1) were calculated for the three isomers using the GAUSSIAN 09 suite of programs^[14]^ and the microcanonical Variational Transition State Theory (μVTST).^[15]^ Vibrational and rotational data obtained for the ionic ground state at the B3LYP/6‐311++G** level of theory (for details see SI) were used to calculate the *k*(E) in the energy range from the ionization energy, IE, of the ground state of the molecule to higher energies according to the equation derived under the rotational adiabatic approximation:

$$k\left(E\right)=\frac{\sigma N^{\neq }\left(E-E_{0}\right)}{h\rho {\left(E\right)}^{ion}}\frac{Q_{rot}^{\neq }}{Q_{rot}^{ion}}\;\left(1\right)$$

where σ is the reaction symmetry factor, N^≠^(E–E_0_) is the sum of the vibrational states of the transition state (TS) at E_0_, either localized on the top of the barrier, wherever it is present (TS1 and TS2 in 2NIM, Figure 1), or by variational minimization of N^≠^ Q^≠^_rot_ along the reaction coordinate, ρ(E)^ion^ is the density of vibrational states of the parent ion at the energy E, Q^≠^_rot_ and Q^ion^_rot_ are rotational partition functions of TS and parent ion respectively, and h is the Planck constant. The dynamics of the NO_2_ loss dissociation channel from 4 and 5NIM isomers, where the barriers were not present, have been explored with density functional theory (DFT), to investigate the minimum energy path (MEP) of the dissociation. The results of these calculations are exploited by VTST, specifically used for barrierless processes,^[16]^ to search for the “bottleneck” of the reaction, namely the Variational Transition State (VTS) configuration at which the reaction flow is at the minimum. The VTS configurations have been obtained at different energies through the minimization of N^≠^(E)Q^≠^_rot_(298.15) along the MEP. The MEPs of 4 and 5NIM which leads to 67^+^ fragment are shown in Figure S2 of SI.

The *k*(E) curves (Figure 3) show the behavior of a *structural rearrangement versus simple bond fission*,^[17]^ with the latter dominating at high energy. In the case of 5NIM the *k*(E) curves cross each other at 12.74 eV, while for 4 and 2NIM the crossing occurs at higher energies: 15.03 and 19.16 eV respectively (data not shown). The results are even clearer once the branching ratio BR=*k*_83+_/[*k*(_83+_+*k*_67+_)] and *k*_67+_/[*k*(_83+_+*k*_67+_)] for the two channels are calculated and reported versus energy for the three isomers (Figure 4).

**Figure 3:**
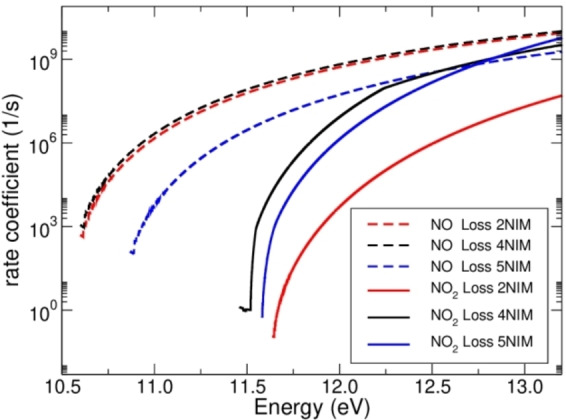
Calculated *k*(E) for the NO_2_ (full lines) and NO loss channels (dashed lines) for the three nitroimidazole isomers, as indicated in the legend.

**Figure 4:**
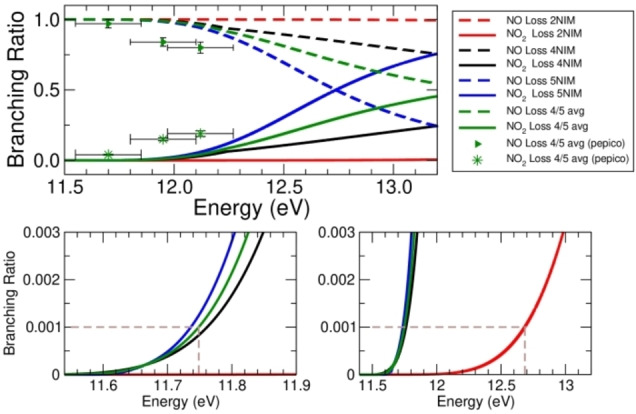
Top panel: branching ratio for the NO_2_ (full lines) and NO loss (dashed lines) channels for the three nitroimidazole isomers. The green lines are obtained by considering that experimentally the 4NIM and 5NIM coexist in a ratio of about 1 : 0.7 in favor of 4NIM.^[10]^ Bottom panel: enlarged view of the top panel in the regions of AE of fragment 67^+^, on the left for 4(5)NIM mixture, on the right for 2NIM. The data points with error bars are the experimental BR from PEPICO spectra (see text) for the two channels in the 4(5)NIM mixture.

A BR of 0.001 can be empirically considered the minimum detection limit for ion 67^+^ from its experimental AE of the NIM isomers.^[8b]^ At this BR the corresponding AE_th_ are 11.75 eV for 4 and 5NIM, and 12.68 eV for 2NIM, showing that in the latter case there is an intrinsic kinetic shift with respect the other two isomers. Figure 4 shows how the BR for the NO loss is constant at about 1.0 in 2NIM, while it decreases in the other isomers, with 5NIM having the steepest fall. On the contrary, the BR for NO_2_ loss is absent in 2NIM, while it rapidly increases in the case of 5NIM. Accordingly, in 2NIM the *kinetic competition* definitely favors the NO loss in the energy range from ionization threshold up to about 12.6 eV where the BR is at the empirical detection limit of 0.001.

As a matter the fact, the *kinetic competition* is clearly visible at the specific energy of 11.75 eV (BR=0.001 for the 4(5)NIM mixture), where the theoretical analysis (Figure 3) finds two very different rate constants in the case of 2NIM: *k*(11.75 eV)=2.05×10^8^ s^−1^ and *k*(11.75 eV)=32.2 s^−1^ for the NO and NO_2_ losses, respectively.

The branching ratios have been also experimentally estimated from the Photoelectron‐Photoion Coincidence (PEPICO) spectra of 2NIM and of the mixture 4(5)NIM.^[8b]^ The experimental BR (see SI) of the parent ion, 113^+^, and of the sum of all ions that originate from fragments 83^+^, the so‐called ‘83^+^ family’:^[8b]^ NO^+^ (30^+^), HCNH^+^ (28^+^), HNC(H)NCH^+^ (55^+^ only from 5NIM), HNC(H)CO^+^ (56^+^ only from 2NIM), and 67^+^ are shown in Figure 5 in the energy range 9.5–12.4 eV. From Figure 5 it is evident that while in 4(5)NIM the production of 67^+^ takes off at about 11.6 eV, in the case of 2NIM the BR of 67^+^ is zero.

**Figure 5:**
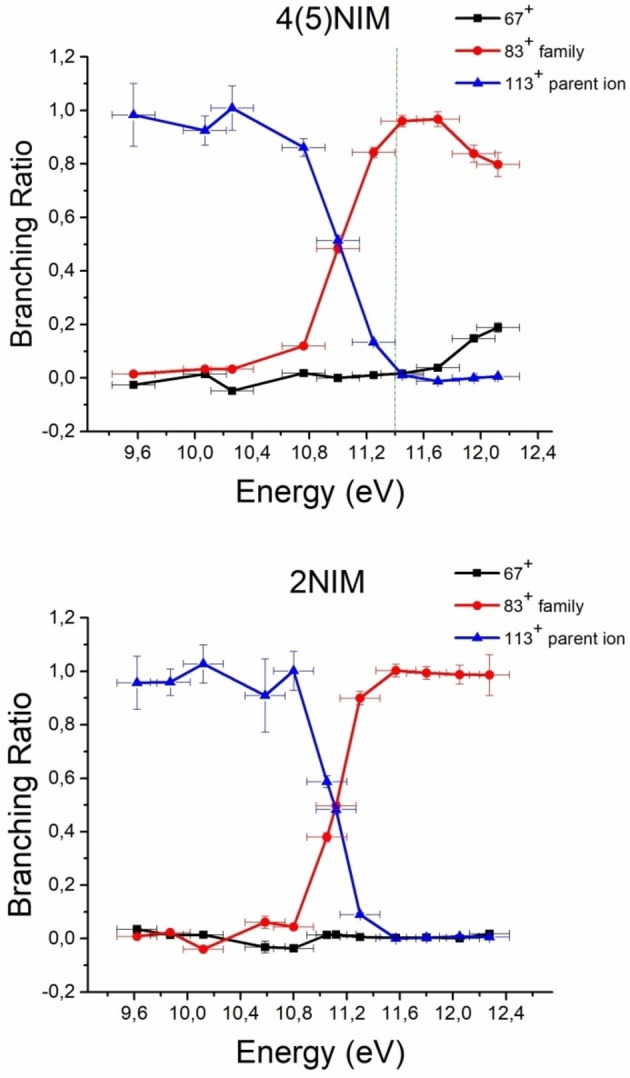
The experimental BR for the parent ion 113^+^, ion 67^+^ and for the sum of all ions that originate from fragment 83^+^, the so‐called 83^+^ family, respectively, for the 4(5)NIM mixture (top panel) and 2NIM (bottom panel), see text.

The calculated BR does not consider theion 113^+^, however the parent ion does not contribute to the PEPICO experimental BR at energies higher than 11.4 eV, thus a comparison between experiments and theory can be attempted for the 4(5)NIM mixture (see green points in Figure 4). An overall satisfactory agreement is observed, and the small discrepancies may be due to non‐statistical energy redistribution of the internal energy of the ions in the experiments, to the possible overestimation of the energy barrier in the theoretical calculations or to the harmonic approximation and vibrational frequencies evaluation.

In summary, the puzzling discrepancy of about 1.0 eV between the theoretical and experimental AEs of fragment 67^+^ in 2NIM has been explained as due to the *kinetic competition* between the NO and NO_2_ loss channels that favours the former and finally explains the lack of observation of ion 67^+^ at energies close to the AE_th_. Furthermore, our calculations suggest that the factors making the NO_2_ loss pathway slower in 2NIM than in 4(5)NIM are to be searched in the structure of TS and VTS of the dissociation process at energies close to the AEs of ion 67^+^ of 4(5)NIM, i. e., 11.75 eV, as shown in Figure 6.

**Figure 6:**
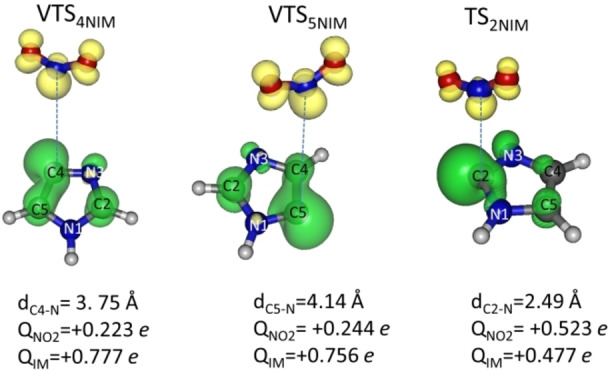
Structures of VTS and TS calculated at the B3LYP/6‐311++G** level of theory for the NO_2_ loss channel in the three isomers. The distance, d, of the broken bond C−N, the Mulliken charge Q in the NO_2_ and imidazole moieties (IM) and the spin isosurface (isovalue=0.010) are also reported.

The comparison of these TS and VTS structures (Tables S5 to S7) in the three isomers shows that only in 2NIM the NO_2_ group still substantially interacts with the imidazole ring, being the C2−N distance equal to 2.49 Å, with the positive Mulliken charge equally distributed on the two moieties. This implies that the vibrational frequencies in the low‐energy range (those associated with the inter‐fragments modes) of the TS of 2NIM are higher than those of 4 and 5NIM. Hence, because N^#^(E) increases more rapidly when the systems have frequencies with lower wavenumbers, the N^#^(E) of 4 and 5NIM increases more rapidly than for 2NIM. This makes the dynamics of the fragmentation in 2NIM slower than in the 4 and 5NIM isomers and the NO_2_ loss channel not competitive with the NO loss in the low energy region.

In conclusion this work i) demonstrates why the NO_2_ loss from 2NIM is observed experimentally only 1.1 eV above its theoretically predicted AE; ii) highlights the importance of the kinetic studies in the fragmentation processes of nitroimidazoles and the competition among the different channels, even when no reaction barrier exists;^[13]^ and iii) explains why 2NIM is a more efficient radiosensitizer than the other isomers, being an efficient NO source, the vasodilator that favors the reoxygenation of hypoxic tissues.

## Conflict of interest

The authors declare no conflict of interest.

## Supporting information

As a service to our authors and readers, this journal provides supporting information supplied by the authors. Such materials are peer reviewed and may be re‐organized for online delivery, but are not copy‐edited or typeset. Technical support issues arising from supporting information (other than missing files) should be addressed to the authors.

Supporting Information
