# Supplementary material for: Ionization of 2‐ and 4(5)‐Nitroimidazoles Radiosensitizers: A “Kinetic Competition” Between NO2 and NO Losses
Source: Chemphyschem. 2021 Oct 12;22(23):2387–91. doi: 10.1002/cphc.202100629 (PMC9293481; doi:10.1002/cphc.202100629)
Supplement: Supplementary file 1 — Supporting Information [file CPHC-22-2387-s001.pdf]

ChemPhysChem

Supporting Information

Ionization of 2- and 4(5)-Nitroimidazoles Radiosensitizers: A *"Kinetic Competition"* Between NO_2 and NO Losses

Mauro Satta,* Anna Rita Casavola, Antonella Cartoni,* Mattea Carmen Castrovilli, Daniele Catone, Jacopo Chiarinelli, Stefano Borocci, Lorenzo Avaldi, and Paola Bolognesi

Supplementary Information

Table of Contents:

I. Table 1S………2S

II. Vectors of the calculated dipole moments of the neutral and ionized NIMs (Figure 1S)………………3S

III. Theoretical details………3S

IV. MEP (Figure 2S)……4S

V. Calculation of Branching Ratio (BR) from PEPICO measurements………………………………………………4S

VI. Table 2S to 10S: geometries at the B3LYP/6-311++G** level of theory………………………………………4S

Table 1S. Theoretical parameters at the B3LYP/6-311++G** level of theory of the neutral and ionic nitroimidazole isomers. Bond length in *Å*, ΔH° and ΔG° in *Hartree*, Q in *e* and dipole moment in *D*.

	Bond Length $C-NO_2$	ΔH°	ΔG°	Q_{NO2}	Dipole
2NIM	1.45	-430.760607	-430.798179	-0.287	5.85
$2NIM^+$	1.46	-430.405719	-430.445089	0.001	8.80
4NIM	1.45	-430.762525	-430.800375	-0.254	7.79
$4NIM^+$	1.45	-430.411460	-430.450586	0.098	9.10
5NIM	1.42	-430.763285	-430.800892	-0.312	3.80
$5NIM^+$	1.45	-430.404071	-430.443611	0.017	5.14

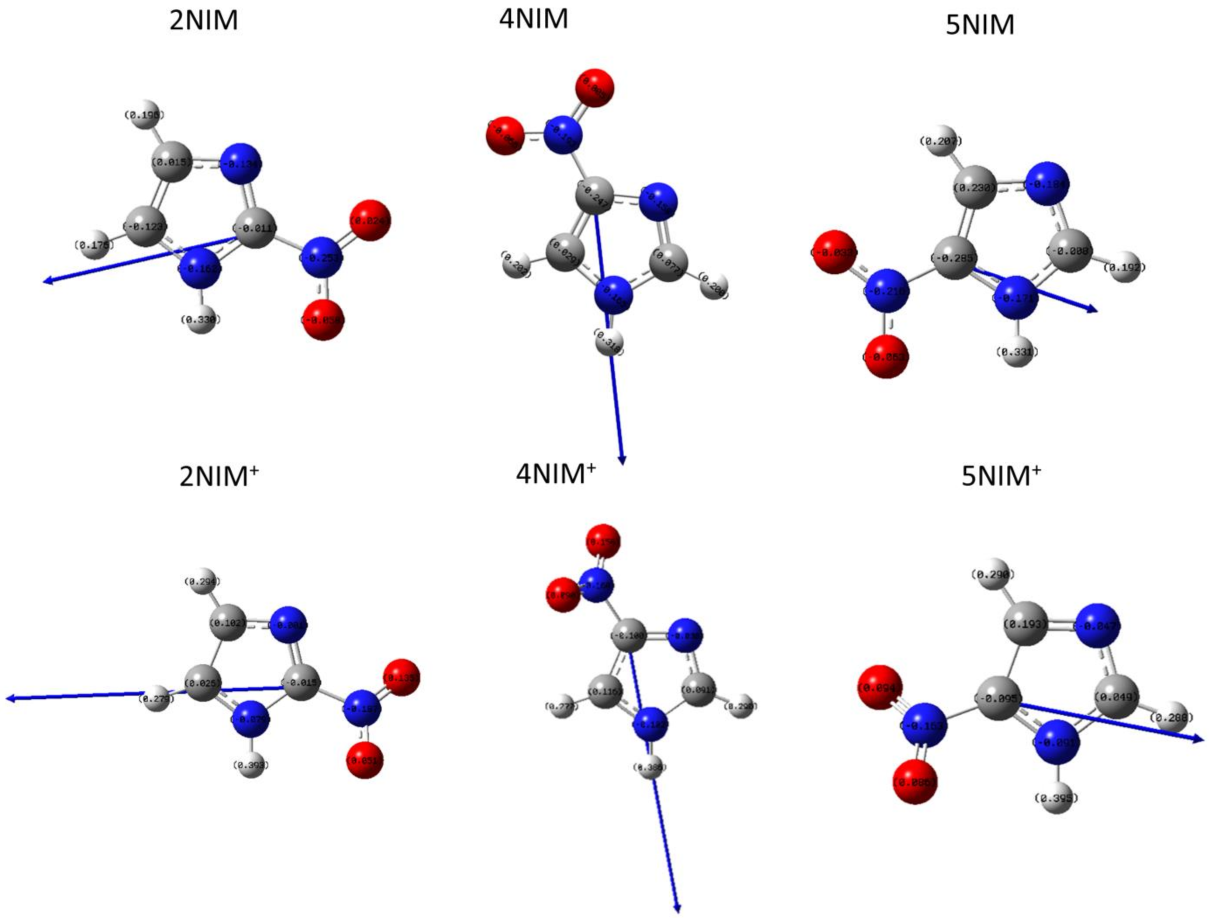

Figure 1S. Vectors of the calculated dipole moments of neutral and ionized 2NIM, 4NIM, 5NIM at the B3LYP/6-311++G** level of theory.

Theoretical details

The Minimum Energy Paths relative to the NO_2 loss for 4 and 5NIM have been calculated at the B3LYP/6-311++G** level of theory: the step along the dissociation coordinate has been kept fixed at 0.1 Å, the frequencies have been calculated in harmonic approximation. The microcanonical rate coefficients have been calculated with the hypotheses that the rotational and translational modes were uncoupled to the vibrational degrees of freedom, and that their energy content can be described by a Boltzmann distribution, so that the rotational molecular partition functions have been used. The geometry of the VTS was obtained minimizing the vibrational number of states along the MEP, without counting the vibrational frequency associated with the reaction coordinate, calculated with direct count Beyer-Swinehart algo-

rithm.[1] The same algorithm has been applied to compute the vibrational density of states of the ion in its ground state minimum energy geometry.

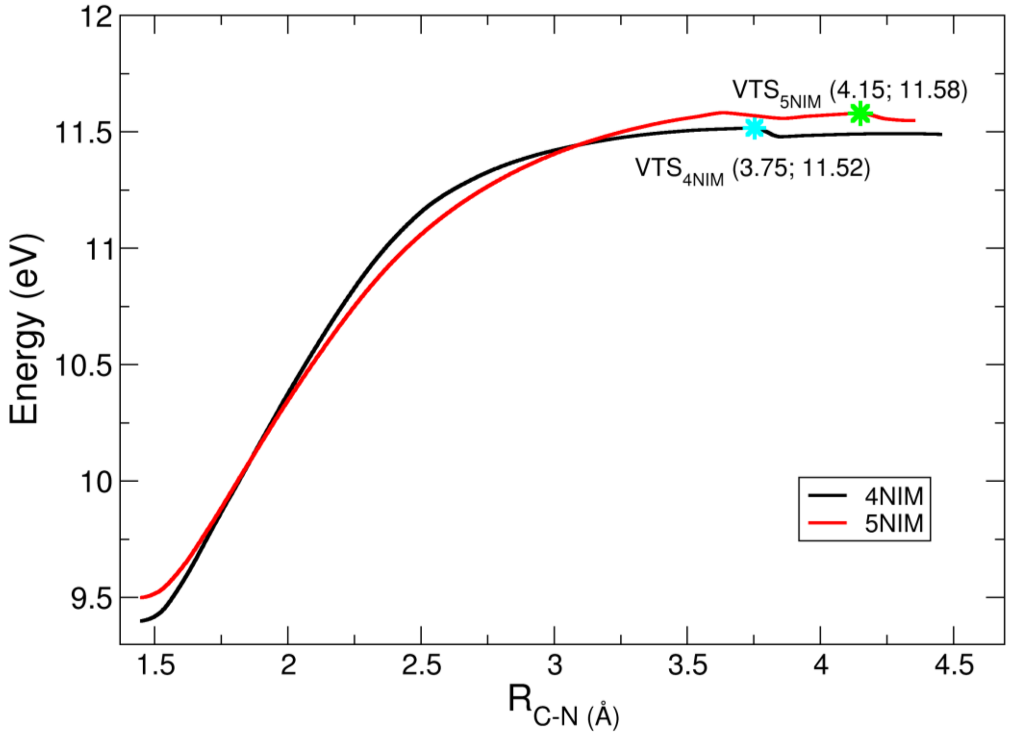

Figure 2S. Minimum energy path (MEP) calculated at the B3LYP/6-311++G** level of theory for the NO_2 loss channel from 4NIM (black line) and 5NIM (red line). The Variational Transition States are shown with light blue (4NIM) and green (5NIM) stars along the MEP. The numbers in brackets indicate, respectively, the C-N distance of the broken bond in Å and the energy of VTS in eV.

Calculation of Branching Ratio (BR) from PEPICO measurements

In a PhotoElectron-PhotoIon COincidence (PEPICO) experiment a mass spectrum is measured detecting in coincidence fragment ions and energy resolved photoelectrons. The kinetic energy (E_k), of the photoelectron selects a molecular ion state whose binding energy (BE) is defined, within the experimental uncertainty, by the difference between the incident photon energy (hv) and E_k. The data reported in Figure 5 of the main text derive from the PEPICO measurements [2] collected at the fixed photon energy of 60 eV, in a BE range from just below the ionization threshold up to 17-18 eV. The experimental resolution is $\Delta E \approx 300$ meV.

Each selected PEPICO spectrum ('labelled' by a specific BE ± ΔE) has been analyzed in the following way to produce data for Figure 5:

i) The area of each m/z peak has been calculated by adding up the ion yield in the region m/z ± 0.5 ;

ii) to extract the branching ratio (BR) of each fragment (or group of fragments) at a selected BE the area (or the sum of the areas of the group of fragments) has been normalized to the sum of the areas of all fragments observed in the PEPICO spectrum.

---

[1] T. Beyer, D. F. Swinehart, Commun. of the ATM 1973, 16, 379

[2] Bolognesi, P.; Casavola, A. R.; Cartoni, A.; Richter, R.; Markus, P.; Borocci, S.; Chiarinelli, J.;Tošić, S.; Sa'adeh, H.; Masič, M. et al. J. Chem. Phys. **2016**, *145*, 191102-1-191102-5.

Table 2S. Cartesian coordinates (Å) for the ion of 2NIM

N	0.005223	0.016967	-0.002340
C	-0.010561	-0.019605	1.386506
N	1.175265	0.158786	1.933139
C	2.007531	0.309434	0.904152
C	1.271417	0.226764	-0.353415
H	-0.813022	-0.052965	-0.605699
H	3.071355	0.469635	1.022866
H	1.604206	0.315638	-1.379422
N	-1.271961	-0.223419	2.101540
O	-1.226422	-0.819633	3.146345
O	-2.238991	0.256802	1.522881

Table 3S. Cartesian coordinates (Å) for the ion of 4NIM

N	0.012253	-0.046835	-0.000591
C	0.011905	0.120277	1.384138
N	1.242772	0.118602	1.877878
C	2.024591	-0.064193	0.827289
C	1.278768	-0.161278	-0.402627
H	-0.810325	-0.082907	-0.597776
H	-0.899151	0.225689	1.958270
H	1.598290	-0.302753	-1.425672
N	3.475072	-0.037921	0.885677
O	3.925473	0.729093	0.029804
O	4.076012	-0.681135	1.699300

Table 4S. Cartesian coordinates (Å) for the ion of 5NIM

N	-0.006573	-0.060729	0.003036
C	0.005742	-0.062420	1.382443
N	1.250270	0.033986	1.875207
C	2.063912	0.118493	0.838021
C	1.271405	0.068643	-0.378004
H	-0.818198	-0.084510	-0.612783
H	-0.900804	-0.118351	1.971896
H	3.141546	0.193931	0.904701

N	1.687300	0.052717	-1.766248
O	0.946893	0.561283	-2.580121
O	2.754192	-0.512970	-1.943221

Table 5S. Cartesian coordinates (Å) for the NO_2 loss TS of 2NIM

N	1.635050	-0.903537	0.543777
C	0.515832	-0.970929	-0.201514
N	0.326487	0.087617	-0.956120
C	1.323141	0.956976	-0.620694
C	2.168773	0.348076	0.301648
H	2.006434	-1.619092	1.157198
H	1.413354	1.931833	-1.073498
H	3.094124	0.676309	0.751060
N	-1.721661	0.060831	0.169721
O	-2.413102	-0.609673	-0.463198
O	-1.616836	0.896153	0.961568

Table 6S. Cartesian coordinates (Å) for the NO_2 loss VTS(11.52eV) of 4NIM

C	0.000000	0.000000	0.000000
N	0.000000	0.000000	1.348844
C	1.317784	0.000000	1.781361
N	2.179184	-0.007141	0.754850
C	1.485865	-0.026297	-0.347706
N	3.706796	2.467110	-2.058579
O	4.088938	3.194900	-1.210983
O	3.921740	2.145349	-3.172981
H	-0.820527	-0.001829	1.946785
H	1.597561	-0.001493	2.825881
H	-0.887378	-0.008370	-0.612555

Table 7S. Cartesian coordinates (Å) for the NO_2 loss VTS(11.58eV) of 5NIM

C	1.788533	-1.014010	-0.671467	0.102853
N	2.412991	0.168380	-0.647167	-0.077474
C	1.862506	0.864362	0.409721	-0.025116
N	0.923751	0.145395	1.031639	-0.052040
C	0.832028	-1.029689	0.402161	-0.103142
N	-2.189401	0.051676	-0.164539	0.011503
O	-3.077100	-0.696020	0.021170	0.138283
O	-1.977803	1.198309	-0.333401	0.093665
H	3.148159	0.478385	-1.275414	0.389459
H	2.169385	1.864295	0.683817	0.257720
H	0.191887	-1.843129	0.707420	0.264227

Table 8S. Cartesian coordinates (Å) for the NO loss TS of 2NIM

C	1.903537	-0.861515	-0.075762
C	2.081506	0.541717	-0.250813
N	0.847104	1.089486	-0.121434
C	-0.009125	0.027790	0.133232
N	0.629468	-1.152516	0.171274
N	-1.505624	0.087208	-0.190014
O	-1.254951	0.236376	1.096847
O	-2.482163	-0.236619	-0.739651
H	0.607500	2.073975	-0.162413
H	2.671160	-1.621619	-0.114391
H	2.966098	1.132388	-0.439492

Table 9S. Cartesian coordinates (Å) for the NO loss TS of 4NIM

N	1.978878	0.694638	-0.123573
C	1.946860	-0.706246	-0.221961
N	0.750559	-1.191982	-0.071652
C	-0.042219	-0.095241	0.133805
C	0.732798	1.124349	0.127342
H	2.804238	1.280678	-0.215657
H	2.843330	-1.284239	-0.409885
H	0.441279	2.157457	0.258325
N	-1.535847	-0.147353	-0.179707
O	-2.490287	0.209696	-0.777388
O	-1.293289	-0.156969	1.121965

Table 10S. Cartesian coordinates (Å) for the NO loss TS of 5NIM

N	0.829501	-1.088296	-0.088713
C	2.057832	-0.515871	-0.183412
N	2.012471	0.824999	-0.116473
C	0.745954	1.156502	0.058930
C	-0.065327	-0.052324	0.088556
H	0.613787	-2.079468	-0.115787
H	2.962367	-1.097316	-0.304149
H	0.382667	2.172073	0.145534
O	-1.277619	-0.101065	1.161881
N	-1.510663	-0.178957	-0.149265
O	-2.435974	0.172395	-0.790491
